# The Summer of Our Discontent: Facing the Challenge of Increasing Heat-Related Illness

**DOI:** 10.31486/toj.23.5039

**Published:** 2023

**Authors:** Kevin Conrad

**Affiliations:** Department of Internal Medicine, Ochsner Clinic Foundation, New Orleans, LA

The summer of 2023 was brutally hot here in Louisiana as well as around the world. According to scientists at NASA, since official records began in 1880, the summer of 2023 was the hottest on Earth by a wide margin.^1^ Correspondingly, the 2023 summer in Louisiana was the hottest on record in the state, with 69 deaths directly attributed to the heat.^2^ Across the nation, emergency services and health care systems were strained. Many places, despite being long accustomed to scorching seasonal heat, were ill prepared. Emergency rooms in Arizona were filled to pandemic levels with patients with heat-related illnesses. Health officials, community leaders, and the general public now recognize heat as a singular, significant cause of both mental and physical harm.

The unprecedented heat had far-reaching impacts, resulting in droughts, crop failures, and deadly wildfires from Canada to Hawaii. Climatologists suggest that this pattern of weather is not an isolated event and will continue, with 2024 predicted to be as hot or hotter than 2023.^3^ Extreme temperature events are increasing in frequency, duration, and magnitude,^3^ and current public health systems are not prepared.

Among weather-related natural disasters, heat causes the most fatalities,^4^ but public health planning and communitywide interventions remain limited. Agreement is limited even on the parameters for a significant heat event to be considered a heat wave.^5^ What is considered extreme heat for one region may not apply to another. One challenge with not having a standard definition for heat waves is that it becomes difficult to determine whether an adverse health presentation occurred within or because of a period of extreme heat.

The impact of heat on a region is determined by many factors, such as local adaptation of the population, housing design, and socioeconomic status. In comparison with other disasters such as earthquakes, hurricanes, and floods, heat events occur gradually rather than causing sudden and predictable damage. Determining a specific start and endpoint is difficult, undermining efforts to plan and to mitigate damage.

As of 2023, only a few US cities—Los Angeles, Miami, and Phoenix—have dedicated chief heat officers to coordinate efforts.^6^ Chief heat officers examine and provide solutions for the increasing impact of heat on health and economic conditions. As this effort typically crosses many aspects of government, chief heat officers facilitate working relationships across all departments within their jurisdiction. A particular area of focus is marginalized populations such as the homeless, the elderly, and individuals living in substandard housing.

The extreme temperatures of the summer of 2023 may have been a tipping point. For US citizens, climate change, which until this summer had been a theoretical concept, had a direct impact on their lives. On a daily basis, heat records were broken, changing the lifestyles of many Americans on large scale. Events were canceled at an unprecedented level; athletic practice times were changed; and those most impacted, such as outdoor workers, took precautions. For the first time, a large majority of Americans—71%—believe that climate change is causing a direct harm to people.^7^

Climate change will present many challenges to delivering health care, and the response to heat-related health may be one of the first and greatest challenges. Addressing increases in heat-related illness will require a systematic, multifactorial approach, including coordination with public officials and increased public awareness. In the short term, practical, feasible, and often low-cost interventions at the local level can save lives. In 2023, cooling centers, early warning systems, and wellness checks were successfully implemented in several urban areas. In addition, relatively easy heat mitigation strategies, such as expanding the tree canopy as a component of urban design, are being incorporated into city planning.^8^

Accurately measuring the impact of heat on mortality and morbidity is a difficult task. Increasing emphasis has been placed on identifying overt heat-related diseases such as heatstroke in the field and in emergency rooms. Heat as a cofactor in other diseases, mental health, and crime is only beginning to be extensively recognized and studied. The true impact of heat on morbidity and mortality is debated. The only consensus among health officials and climatologists is that in the United States, heat-related fatalities and morbidity are grossly undercounted.^4,9^

Several studies demonstrate that the increasing temperature is affecting health. According to a 2020 report in *The Lancet*, heat-related mortality among persons older than 65 years of age increased at least 54% in the prior 20 years.^10^ Outdoor workers in the United States have up to 35 times the risk of dying from heat exposure than the general population.^11^

Many diseases are thought to be heat sensitive and many populations are particularly at risk*.* Several studies have demonstrated an increase in the occurrence of a wide range of conditions during periods of high temperature, including asthma, chronic obstructive pulmonary disease, respiratory infections, ischemic heart disease, cardiac dysrhythmias, ischemic stroke, hyperglycemia, kidney failure, and neuropsychiatric disorders.^12-15^ The mental health burden is significant, with increasing ambient temperature resulting in an increase in psychosis, suicides, homicides, anxiety, and depression.^16^ Maternal-fetal health is particularly impacted; heat waves are associated with preterm delivery and small-for-gestational-age infants.^17^

In response to the new normal of increasing ambient temperatures and heat waves, health systems and providers must expand their research, planning, and interventions. This work includes developing a more robust system of capturing the direct and indirect causes of heat on mortality and morbidity, creating early heat-related warning systems for vulnerable populations, and educating clinicians on the impact of heat as a cofactor in disease progression and management. In addition, health systems should coordinate with public agencies that provide services and warnings to at-risk populations, such as the susceptible elderly and their caregivers, pregnant women, outdoor workers, and those engaging in outdoor athletic activities.^18^

Currently, strategies to prevent heat-related illness are directed largely by clinical experience and observational data. Research in this area is limited, and guidelines are only beginning to be developed.^19^ The limited evidence supports screening for those at risk and using behavioral interventions. All clinicians, but especially those in primary care, should identify vulnerable patients and review their medical histories for appropriate interventions, such as reviewing the drugs they are taking and adjusting for side effects and optimum dosing during periods of hot weather. Patients should also be educated on the impact heat may have on their current medical conditions and how to recognize and respond to heat exhaustion.

Adaptation, which may take more than one warm season, is a key factor in heat-related morbidity. Importantly, the usual steps that individuals take in a particular region to combat heat may no longer work in an area that sees only a few degrees of temperature elevation from the norm.

Although public health heat warning systems continue to evolve, work is still needed to identify and alert specific populations. Many at-risk populations, such as the homeless and the elderly, are difficult to inform with typical media resources.^20^

Although models continue to predict global increases in temperature, there are reasons to be optimistic. The general public, health care systems, and governmental agencies are increasingly engaged in efforts to mediate climate change–related issues. With feasible action, heat-related illness can be markedly reduced. We must ensure health care providers and health care system administrators understand the direct impact of heat on illness and the critical role they can play in reducing its damage. Their primary role is to raise awareness among the public and community leaders of the increasing risk of heat-related illness.

This summer of intense heat in 2023 affirms that the era of climate change medicine is here, and traditional health care delivery will need to adapt. As with many population health initiatives, success in dealing with heat-related illness will require health systems to define the problem, provide traditional and nontraditional remedies, and lead efforts across a wide variety of public services.
